# The effect of a digital-assisted group rehabilitation on clinical and functional outcomes after total hip and knee arthroplasty—a prospective randomized controlled pilot study

**DOI:** 10.1186/s12891-023-06270-8

**Published:** 2023-03-14

**Authors:** Judith Osterloh, Franziska Knaack, Rainer Bader, Martin Behrens, Juliana Peschers, Lisa Nawrath, Philipp Bergschmidt, Martin Darowski

**Affiliations:** 1 Department of Orthopaedics, Rostock University Medical Center, Rostock, Germany; 2 Department of Sport Science, Institute III, Otto-Von-Guericke University Magdeburg, Magdeburg, Germany; 3 Vital & Physio GmbH, Rostock, Germany; 4 Clinic for Orthopaedics, Trauma and Hand Surgery, Klinikum Südstadt Rostock, Rostock, Germany

**Keywords:** Total knee replacement, Total hip replacement, Endoprosthesis, Postoperative rehabilitation, Physiotherapy, Digitalization, Monitor-based therapy

## Abstract

**Background:**

The rising number of total hip and knee arthroplasties and the decreasing availability of physiotherapists require clinically and economically effective rehabilitation approaches. Therefore, the present pilot study investigated the effect of a novel digital-assisted individualized group rehabilitation program on clinical and functional outcomes after total hip and knee arthroplasty.

**Methods:**

In this randomized controlled pilot study, 26 patients undergoing total knee or hip replacement were randomly assigned to either the intervention group (IG, novel digital-assisted group therapy) or the control group (CG, standard postoperative physiotherapy currently carried out in Germany). The IG received the novel digital-assisted group therapy twice per week for a six-months period, while the CG received individual outpatient therapy depending on the prescription of the supervising physician. The number of therapy sessions was recorded. Moreover, subjective outcomes (EuroQol-5Dimensions (EQ-5D) and Western Ontario and McMaster Universities Osteoarthritis Index (WOMAC)), functional outcome (30 s sit to stand test and timed up and go test (TUG)), as well as gait parameters were quantified preoperatively as well as at three and six months after surgery. Data were analyzed using an analysis of covariance with baseline-adjustment.

**Results:**

No patient-reported falls, pain, and hospital readmissions were recorded. On average, the IG received more therapy sessions. The clinical and functional outcomes were mainly not significantly different between groups at three- and six-months follow-up, but medium to large effect sizes for the differences in quality of life (EQ-5D) as well as pain, stiffness, and physical function (WOMAC), and TUG performance were observed in favor of the IG. However, the IG showed a higher variability of gait velocity after total joint replacement.

**Conclusion:**

The digital-assisted rehabilitation had positive effects on quality of life, pain, stiffness, physical function, and TUG performance. Nevertheless, the therapy concept may be improved by adding exercises focusing on gait performance to reduce gait variability. The results indicate that the digital-assisted therapy concept is effective and safe. Therefore, a consecutive full-scaled randomized controlled clinical trial is recommended.

**Trial registration:**

This study was retrospectively registered on 14/02/2022 in the German Clinical Trial Register (DRKS00027960).

**Supplementary Information:**

The online version contains supplementary material available at 10.1186/s12891-023-06270-8.

## Background

Osteoarthritis (OA) of the knee and hip are two of the major global medical conditions. A study examining the current health status of German citizens showed that the prevalence for OA in the group of 45- to 64-year-old German citizens was 23.3% for females and 16.6% for males. In the over 65 age group, half of the females (48.1%) and one third of the males (31.2%) were already affected by OA [1, 2]. A similar prevalence for radiographic OA of the knee or hip joint was reported for elderly US citizens. Knee or hip OA lead to individual disability, reduced quality of life and have a substantial socioeconomic impact [3, 4]. In the end-stage of hip and knee OA, joint replacement is the preferred treatment to mitigate pain and restore mobility. Among other factors, demographic changes led to a rising rate of total knee and total hip arthroplasties in the last years [5]. Clinical outcomes, especially after total knee arthroplasty, are not always optimal and new and effective rehabilitation approaches are required [6]. Although physiotherapy in general shows a clinical benefit after total knee and hip arthroplasty, there is a need for cost-efficient rehabilitation strategies that could help to improve medium- and long-term outcomes [7, 8]. To this day, a sufficient physiotherapy support after discharge is facing several problems. For example, the access to physiotherapy providers, especially for patients in rural areas, is often limited [9]. Furthermore, the modern society is confronted with an increasing shortage of physiotherapeutic specialists [10–12]. Therefore, it is crucial to improve current physiotherapy concepts by providing cost- and time-efficient rehabilitation approaches. In current scientific literature, different approaches were discussed to address the above mentioned problems. Some studies investigated the effectiveness of technical solutions for rehabilitation at home and showed that home-based therapy is as effective as normal outpatient physiotherapy [13, 14]. Furthermore, group physiotherapy provides similar outcomes as one-on-one physiotherapy [15] and allows a better distribution of financial resources [16]. However, a disadvantage of group therapies is the lack of individualization of exercises for patients with different conditions. To the best of our knowledge, no rehabilitation concepts exist, which have combined the advantages of an individualized therapy with the cost and time effectiveness of group therapy by using a digital-assisted approach for knee and hip arthroplasty patients. Therefore, the aim of this pilot study was to assess the effect of a novel digital-assisted individualized group therapy concept on subjective outcomes (EuroQol-5Dimensions (EQ-5D) and Western Ontario and McMaster Universities Osteoarthritis Index (WOMAC)), strength of the lower extremities (30 s sit to stand test (STS)), and functional mobility (timed up and go test (TUG)), as well as gait parameters (gait velocity, step length, and the coefficient of variation (CV) of these parameters).

## Materials and Methods

### Study Design

A prospective randomized controlled pilot study with a two-arm parallel group design was conducted from August 2020 to December 2021. Approval of the Ethics Committee of the University Medical Center Rostock was obtained (A 2019–0211). The Consolidated Standards of Reporting Trials Statement (CONSORT) for randomized pilot trials was used for reporting [17]. All methods were carried out in accordance with relevant guidelines and regulations of the declaration of Helsinki. This pilot study was retrospectively registered on 14/02/2022 in the German Clinical Trial Register (DRKS00027960).

### Participants

Participants were recruited preoperatively at the Department of Orthopaedics at the Rostock University Medical Center and the Clinic for Orthopaedics, Trauma and Hand Surgery at the Klinikum Südstadt Rostock. Only males and females within the age range of 55 to 70 years, requiring unilateral total hip or knee arthroplasty due to primary or secondary OA were included in the study. Furthermore, only participants who were able to participate in the outpatient therapy during the first postoperative year as well as the required follow-up examinations were included. Patients with cachexia or obesity (body mass index (BMI) < 19 kg/m^2^ or > 35 kg/m^2^) or severe systemic disease (American Society of Anesthesiologists score > 2) were excluded. In addition, acute or suspected infections and the presence of severe heart failure or chronic obstructive pulmonary disease resulted in exclusion from the study. The same applied to patients with a neurological disease affecting cognition as well as to patients with acute spinal diseases or joint, muscle or systemic diseases affecting gait. Eligible patients gave their informed consent and were randomly assigned to either the intervention group (IG, i.e., novel group therapy concept with video-based rehabilitation tool) or the control group (CG, i.e., standard physiotherapy currently carried out in Germany after total knee and hip arthroplasty) using block randomization (allocation ratio of 1:1). Group assignment was performed sequentially according to computer-generated allocation order, but group affiliation was only shared with the patient after consent to participate. This pilot study was conducted to generate preliminary estimates of effectiveness of the digital-assisted individualized rehabilitation approach. Therefore, no a priori sample size calculation was carried out. Based on the number of previous knee and hip arthroplasty surgeries of both participating clinics and according to the inclusion and exclusion criteria, it was assumed that a sample size of 30 patients could be achieved in the intended time frame. Blinding of patients, physical therapists, and investigators was not possible because of the nature of the intervention.

### Interventions

**Fig. 1:**
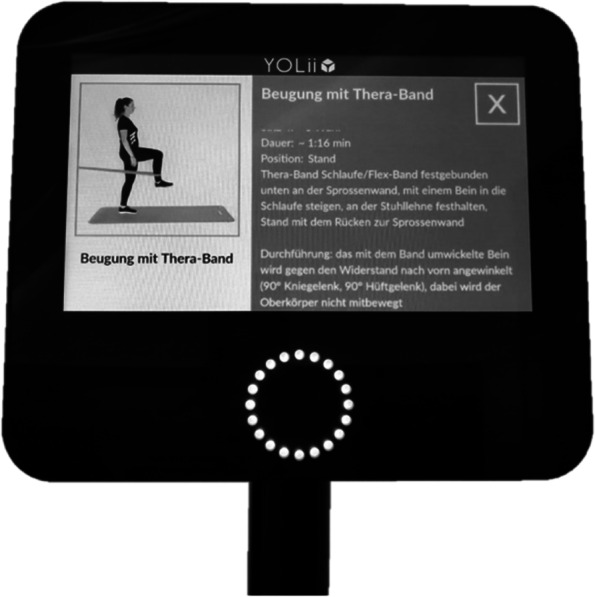
YOLii monitor showing exercise instruction and explanatory video

Both recruiting medical centers are certified endoprosthesis centers of maximum care (EPZmax, EndoCert GmbH, Berlin, Germany). In total, eight experienced orthopaedic surgeons were involved in this study and for each surgery, one of the EndoCert certified surgeons was present. After surgery and inpatient care in the hospital, all participants (IG and CG) underwent three to four weeks of standard inpatient or outpatient rehabilitation. Afterwards, the IG received outpatient therapy twice a week until the sixth postoperative month. During this period, the participants received standardized group therapy with the video-based rehabilitation tool YOLii (E + S Gesunde Lösungen GmbH, Hamburg, Germany), which was supervised by a physiotherapist. Every participant had a personalized login to the YOLii system. Based on the ICD-10 codes (International Statistical Classification of Diseases and Related Health Problems), a pre-selection of exercises was suggested to the physiotherapist, which was then individually adapted to the patient’s needs. This allows supervision of up to five patients simultaneously by one therapist. Six warm-up exercises, 24 main exercises, and four stretching exercises were selected in the YOLii system based on the recommendations of the American Academy of Orthopaedic Surgeons as well as the recommendations of Vancouver General Hospital and Richmond Hospital [18–21]. The exercises were subdivided into four different therapy blocks. The fist block consisted of strengthening exercises using resistance bands or weight cuffs and the second block included exercises for the core muscles using the own body weight. For the third block, a stepper was used for aerobic exercises and the fourth block consisted of balance exercises using a balance pad. Each individual therapy session included two warm-up exercises, four main exercises (one of each block), and two stretching exercises. To ensure an user-friendly therapy, written instructions as well as an explanatory video for each exercise were shown on the YOLii monitor (Fig. 1). A therapy session lasted approximately 30 min with an active exercise time of 20 min. Individual adjustment of the exercise variables (e.g., number of repetitions) was accomplished by using session ratings of perceived exertion [22, 23] after the first therapy session and every third session during the postoperative therapy process. A list of the physiotherapeutic exercises performed during the intervention can be found in the appendix (additional file 1). Patients in the IG were allowed to seek additional therapeutic assistance (e.g., lymphatic drainage) if necessary.

Participants in the CG continued individual outpatient therapy after the three to four weeks standard rehabilitation depending on the prescription of the supervising physician. The number of therapy sessions for both groups was inquired at the six-months follow-up. Nevertheless, the duration of each session was not considered due to the variety of therapy approaches with different therapy durations depending on the doctor's prescription. As the novel digital-assisted group therapy concept is not yet supported by the German health insurance, we were not able to report the costs for both therapy approaches.

### Study Procedure and Outcome Measures

All outcomes were measured at the preoperative assessment, which took place immediately before the surgical intervention, and two postoperative follow-ups at three and six months after surgery. After arrival at the laboratory, participants had to fill in the EQ-5D and WOMAC questionnaires followed by the TUG and STS tests. Subsequently, the gait analysis was conducted.

To assess the quality of life, the German EQ-5D questionnaire was used. According to Schulenburg et al. [24, 25], an EQ-5D index was calculated for the five dimensions (mobility, self-care, usual activities, pain/discomfort, and anxiety/depression) with 100 being the optimal status. Moreover, patients had to rate their general health status on a visual analog scale (0 to 100%). Pain, stiffness, and physical function of the affected joints (knee or hip) were assessed with the WOMAC score. In this study, a German Likert scale version of the WOMAC from zero to four was used and for each dimension (pain, stiffness, function) the sum of all items was taken. Lower WOMAC scores correspond to less pain, stiffness, or functional limitations.

Strength of the lower extremities was measured with the STS test, which has been shown to be a reliable and valid indicator of lower body strength in older adults [26]. During the STS test, the patients’ placed their hands at the hip and were not allowed to use them as support. The number of STS repetitions in 30 s were recorded.

Functional mobility was measured with the TUG test. It is a common test in physiotherapy and clinical trials to measure the walking ability and functional mobility of patients [27, 28]. During the TUG, patients were asked to stand up from a chair and walk as quickly and stable as possible to a three-meter marked line and to return. The time to complete the test was measured using a stopwatch and the use of assistive walking devices was permitted.

Gait parameters were measured using a six-meter electronic walkway (OptoGait, Microgate Corporation, Bolzano, Italy). To ensure a constant walking velocity during the measurement, a two-meter acceleration and deceleration area was given on each end of the walkway [29, 30]. Five consecutive trials were performed and the first and last step of each trial were excluded. The mean of the remaining steps for the left and right leg as well as of all trials was calculated. Gait velocity and step length were analyzed as well as their CV as a measure of gait variability.

### Statistical Analysis

Data of all participants who completed the pre- and postoperative measurements were included in the statistical analysis and were checked for normal distribution with the Shapiro-Wilk test. Differences between groups in the participants characteristics were examined using independent t-tests (age, weight, BMI), Mann-Whitney-U-test (height) or Fisher's exact tests (sex, joint) depending on normal distribution and scale level. The between-group comparison at three and six months after surgery was performed using an analysis of covariance (ANCOVA) with baseline-adjustment [31, 32]. Furthermore, sex, age, height, weight and the location of the replaced joint (hip or knee) were also entered as covariates. Since the number of therapy sessions was only inquired six months after surgery, additional ANCOVAs were conducted for the six-months follow-up data with the number of therapy sessions as a further covariate. The level of significance was set at p ≤ 0.05 and the effect size partial eta squared (η_p_^2^) was calculated (small η_p_^2^ ≥ 0.01; medium η_p_^2^ ≥ 0.06; large η_p_^2^ ≥ 0.14). Due to the small sample size, results were interpreted based on the effect sizes with a medium effect considered as meaningful. Additionally, a post-hoc power analysis was performed (G*Power 3.1.9.7, Heinrich Heine University, Duesseldorf, Germany) to provide data for the calculation of the sample size for a future randomized controlled trial. All data were analyzed using IBM SPSS Statistics version 27 (IBM Corp., Armonk, N.Y., USA). Data are presented as means ± standard deviations and mean differences (95% confidence interval (CI)).

## Results

**Fig. 2:**
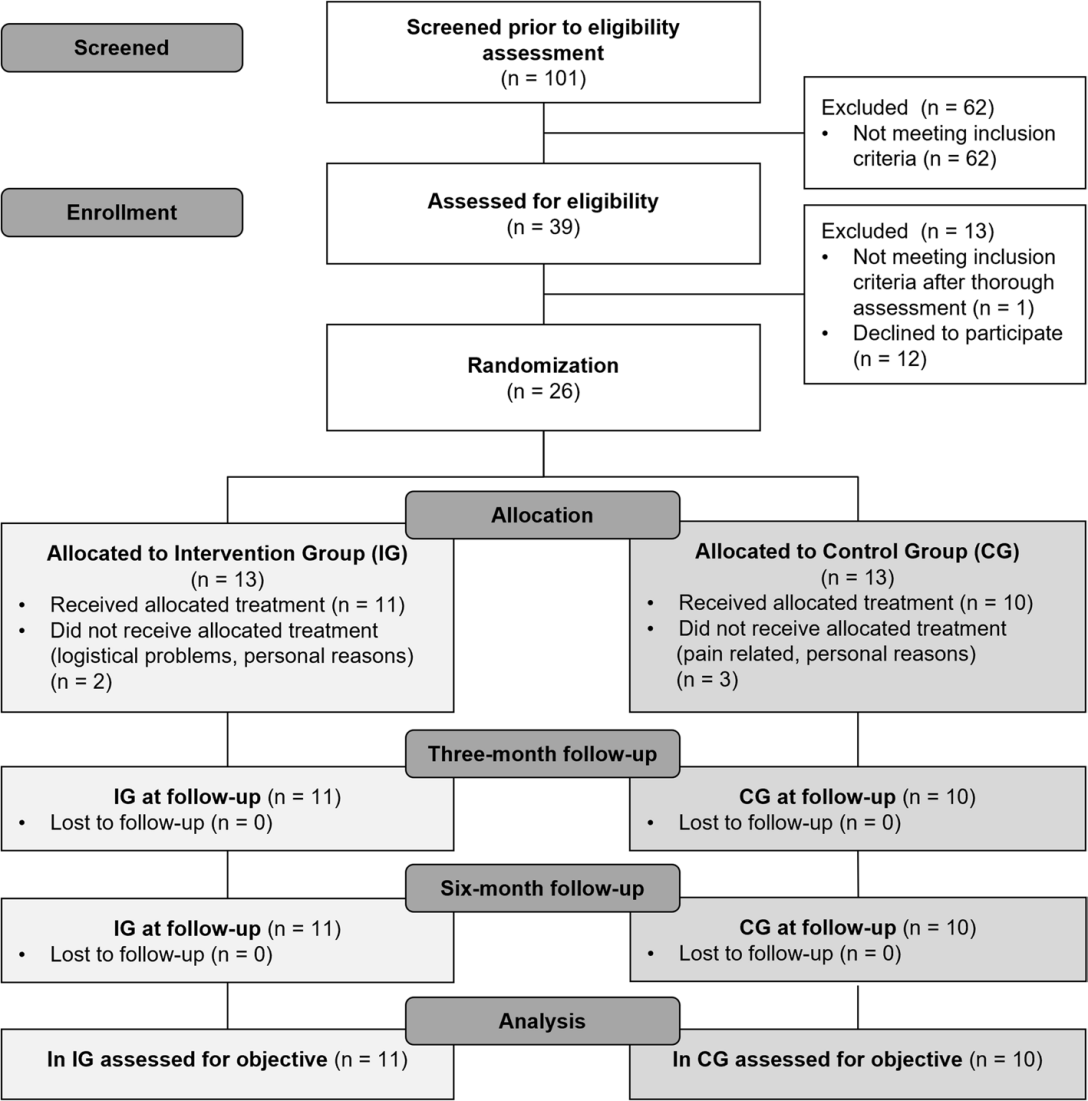
CONSORT flow diagram of progress through phases of this pilot study

From August 2020 till June 2021, a total of 101 individuals were assessed for eligibility. The limitations of elective surgeries and strict hygiene regulations during the physiotherapeutic treatments caused by the COVID-19 pandemic led to a delay of the recruitment and to the expiration of study funding. Therefore, the acquisition goal of 30 participants was not reached. Twenty-six individuals met the inclusion and exclusion criteria and were randomized to either the IG (n = 13) or CG (n = 13). The patient flow diagram is shown in Fig. 2. Due to five dropouts during the follow-up measurements, 11 patients in the IG and 10 patients in the CG were included in the statistical analysis. Overall, no patient-reported falls, pain, and hospital readmissions were recorded. According to verbal inquiry, the IG received significantly more therapy sessions (p = 0.001). Additionally to the digital-assisted group therapy twice a week, patients in the IG required lymphatic drainage or similar treatments shortly after surgery resulting in an average of 39 therapy sessions per patient (32 group and seven individual sessions), while patients in the CG received on average 21 therapy sessions. No significant differences were found between the two groups regarding anthropometric measurements, sex, or affected joint (hip/knee) (Table 1). The results for the preoperative assessments are given in Table 2.

**Table 1:** Participant characteristics for intervention group (IG) and control group (CG)

Parameter	IG	CG	p
Joint (hip—knee)	4—7	6—4	0.395
Sex (f—m)	8—3	4—6	0.198
Age (years)	65.73 ± 2.76	64.00 ± 2.76	0.411
Height (m)	1.72 ± 0.06	1.73 ± 0.08	0.639
Weight (kg)	90.73 ± 11.24	87.00 ± 7.51	0.388
BMI (kg/m^2^)	30.88 ± 4.14	29.09 ± 2.51	0.252
Number of therapy sessions	39.09 ± 8.10	21.20 ± 11.59	0.001*

*Values for each group are shown as means* ± standard deviations; BMI: body mass index; * p ≤ *0.05*

**Table 2:** Preoperative values for intervention group (IG) and control group (CG) and mean differences between groups

Outcome Measures	Preoperative Measurements			
	**IG**	**CG**	**Diff.**	**p**
EQ-5D Index	40.48 ± 17.49	70.78 ± 23.19	-30.31	0.005*
EQ-5D health status (%)	54.05 ± 12.09	66.00 ± 13.50	-11.95	0.045*
WOMAC pain	11.50 ± 2.88	10.75 ± 2.30	0.75	0.528
WOMAC stiffness	5.40 ± 1.35	4.70 ± 1.57	0.70	0.299
WOMAC function	41.30 ± 8.43	33.83 ± 9.33	7.47	0.075
STS (rep)	7.00 ± 4.00	9.11 ± 2.20	-2.11	0.220
TUG (s)	11.67 ± 3.17	10.33 ± 1.47	1.34	0.167
gait velocity (m/s)	1.04 ± 0.15	1.07 ± 0.09	-0.03	0.522
step length (cm)	58.45 ± 6.69	60.60 ± 3.59	-2.16	0.366
gait velocity_CV_ (%)	3.59 ± 1.63	3.18 ± 0.72	0.41	0.475
step length_CV_ (%)	5.94 ± 4.36	6.55 ± 1.70	-0.61	0.132

*Values for each group are shown as means* ± standard deviations; Diff.: mean difference between groups; EQ-5D: EuroQol-5Dimensions; WOMAC: Western Ontario and McMaster Universities Osteoarthritis Index; STS: 30 s sit to stand test; TUG: timed up and go test; CV: coefficient of variation; rep: repetitions; * p ≤ *0.05*

No significant differences between groups were found for all outcomes at three-months follow-up (Table 3). However, medium effect sizes were revealed for the WOMAC stiffness dimension (1.06 (95% CI: -0.70 – 2.82, η_p_^2^ = 0.125)) and the CV of gait velocity (0.95% (95% CI: -0.64 – 2.54%, η_p_^2^ = 0.124)) with higher values in the IG compared to CG.

**Table 3:** Therapy-related group differences after total hip/knee arthroplasty when not accounting for number of therapy sessions

Outcome Measures	Follow-up	n (IG—CG)	IG	CG	Diff. (95% CI)	p	η_p_^2^	Power
EQ-5D Index	3-months	11—10	77.04 ± 17.96	82.65 ± 18.25	-5.60 (-25.33 – 14.13)	0.550	0.028	0.108
	6-months	11—10	85.56 ± 23.65	66.48 ± 24.03	19.09 (-6.90 – 45.08)	0.137	**0.162**	0.462
EQ-5D health status (%)	3-months	11—10	70.84 ± 14.89	73.98 ± 15.08	-3.14 (-19.06 – 12.78)	0.667	0.014	0.078
	6-months	11—10	79.81 ± 11.02	72.11 ± 11.16	7.70 (-4.07 – 19.48)	0.181	**0.133**	0.383
WOMAC pain	3-months	10—10	4.59 ± 2.79	4.71 ± 2.79	-0.11 (-3.13 – 2.90)	0.937	0.001	0.052
	6-months	10—10	3.65 ± 3.38	5.55 ± 3.38	-1.91 (-5.56 – 1.75)	0.278	**0.097**	0.285
WOMAC stiffness	3-months	10—10	3.68 ± 1.63	2.62 ± 1.63	1.06 (-0.70 – 2.82)	0.215	**0.125**	0.343
	6-months	10—10	2.39 ± 1.78	3.21 ± 1.78	-0.82 (-2.74 – 1.10)	0.371	**0.067**	0.207
WOMAC function	3-months	10—10	19.52 ± 8.73	21.31 ± 8.73	-1.79 (-11.39 – 7.81)	0.692	0.014	0.078
	6-months	10—10	13.96 ± 13.25	20.54 ± 13.25	-6.57 (-21.15 – 8.00)	0.345	**0.074**	0.225
STS (rep)	3-months	11—9	10.21 ± 5.13	9.63 ± 5.26	0.58 (-5.10 – 6.26)	0.827	0.004	0.058
	6-months	11—10	10.97 ± 5.60	9.94 ± 5.67	1.03 (-4.94 – 6.99)	0.716	0.011	0.073
TUG (s)	3-months	11—9	9.72 ± 3.24	10.02 ± 3.32	-0.30 (-3.89 – 3.29)	0.860	0.003	0.056
	6-months	11—10	8.05 ± 3.16	9.88 ± 3.20	-1.83 (-5.21 – 1.54)	0.262	**0.096**	0.283
gait velocity (m/s)	3-months	11—9	1.26 ± 0.31	1.24 ± 0.32	0.02 (-0.32 – 0.36)	0.900	0.001	0.052
	6-months	11—10	1.39 ± 0.26	1.31 ± 0.25	0.08 (-0.20 – 0.35)	0.551	0.028	0.111
step length (cm)	3-months	11—9	66.08 ± 6.66	65.04 ± 6.81	1.05 (-6.25 – 8.34)	0.760	0.008	0.066
	6-months	11—10	68.75 ± 6.53	66.77 ± 6.60	1.99 (-2.83 – 9.62)	0.545	0.029	0.113
gait velocity_CV_ (%)	3-months	11—9	3.85 ± 1.37	2.90 ± 1.49	0.95 (-0.64 – 2.54)	0.217	**0.124**	0.341
	6-months	11—10	3.97 ± 1.13	2.44 ± 1.15	1.53 (0.27 – 2.79)	0.021*	**0.347**	0.870
step length_CV_ (%)	3-months	11—9	5.18 ± 1.76	5.02 ± 1.82	0.16 (-1.83 – 2.16)	0.861	0.003	0.056
	6-months	11—10	3.96 ± 1.66	4.30 ± 1.68	-0.34 (-2.13 – 1.45)	0.690	0.013	0.078

*Values for each group are shown as adjusted means* ± standard deviations; n: sample size; IG: intervention group; CG: control group; Diff. (95% CI): mean difference between groups ( 95% confidence interval); EQ-5D: EuroQol-5Dimensions; WOMAC: Western Ontario and McMaster Universities Osteoarthritis Index; STS: 30 s sit to stand test; TUG: timed up and go test; CV: coefficient of variation; rep: repetitions; * p ≤ *0.05; medium (η*_*p*_^*2*^ ≥ *0.06) and large effect sizes (η*_*p*_^*2*^ ≥ *0.14) indicated by bold text*

**Fig. 3:**
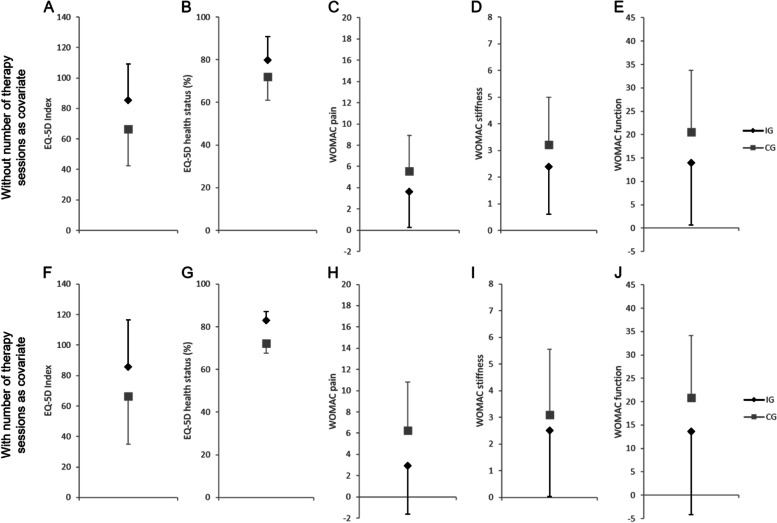
Therapy-related group differences of subjective outcome variables six months after total hip and knee arthroplasty. Values for each group are shown as adjusted means ± standard deviations; A-E: number of therapy sessions is not accounted for; F-J: number of therapy sessions is accounted for as additional covariate; IG: intervention group; CG: control group; EQ-5D: EuroQol-5Dimensions, WOMAC: Western Ontario and McMaster Universities Osteoarthritis Index

At the six-months follow-up, only the CV of gait velocity was significantly different between groups with higher values in the IG (1.53% (95% CI: 0.27 – 2.79%, p = 0.021, η_p_^2^ = 0.347)). However, quality of life was higher in the IG indicated by large effect sizes for the differences in the EQ-5D index (19.09 (95% CI: -6.90 – 45.08, η_p_^2^ = 0.162)) and EQ-5D health status (7.70% (95% CI: -4.07 – 19.48%, η_p_^2^ = 0.133)). Furthermore, medium effects were found for the differences in the WOMAC dimensions indicating that the IG had lower pain (-1.91 (95% CI: -5.56 – 1.75, η_p_^2^ = 0.097)) and stiffness (-0.82 (95% CI: -2.74 – 1.10, η_p_^2^ = 0.067)) as well as a better physical function (-6.57 (95% CI: -21.15 – 8.00, η_p_^2^ = 0.074)). Besides this, a medium effect size was found for the difference in TUG test performance with better values in the IG compared to CG (-1.83 s (95% CI: -5.21 – 1.54 s, η_p_^2^ = 0.096)). The results of the ANCOVA not accounting for the number of therapy sessions are presented in Table 3 and Fig. 3.

When the number of therapy sessions was added to the statistical model as an additional covariate for the six-months follow-up analysis, the significant difference between groups remained for the CV of gait velocity with higher values in the IG compared to the CG (1.80% (95% CI: 0.11 – 3.49%, p = 0.039, η_p_^2^ = 0.309)). Furthermore, the group differences with medium to large effect sizes in favor of the IG remained also for the EQ-5D index (19.39 (95% CI: -17.75 – 56.53, η_p_^2^ = 0.097)), EQ-5D health status (14.24% (95% CI: -2.78 – 31.25%, η_p_^2^ = 0.217)), and WOMAC pain (-3.34 (95% CI: -8.93 – 2.24, η_p_^2^ = 0.136)). Interestingly, step length was higher in the IG with a medium effect (4.69 cm (95% CI: -5.97 – 15.35 cm, η_p_^2^ = 0.071)) (Table 4, Fig. 3). The results of the post-hoc power analysis are presented in Table 5.

**Table 4:** Therapy-related group differences after total hip/knee arthroplasty with number of therapy session as additional covariate

Outcome Measures	Follow-up	n (IG—CG)	IG	CG	Diff. (95% CI)	p	η_p_^2^	Power
EQ-5D Index	6-months	11—10	85.71 ± 30.66	66.32 ± 31.51	19.39 (-17.75 – 56.53)	0.278	**0.097**	0.282
EQ-5D health status (%)	6-months	11—10	82.92 ± 4.25	68.69 ± 4.57	14.24 (-2.78 – 31.25)	0.093	**0.217**	0.601
WOMAC pain	6-months	10—10	2.93 ± 4.55	6.27 ± 4.55	-3.34 (-8.93 – 2.24)	0.215	**0.136**	0.387
WOMAC stiffness	6-months	10—10	2.51 ± 2.47	3.10 ± 2.47	-0.59 (-3.63 – 2.45)	0.677	0.016	0.084
WOMAC function	6-months	10—10	13.63 ± 17.75	20.87 ± 17.75	-7.23 (-29.05 – 14.58)	0.481	0.046	0.153
STS (rep)	6-months	11—10	10.45 ± 2.30	10.50 ± 2.48	-0.05 (-9.30 – 9.19)	0.990	0.000	n/a
TUG (s)	6-months	11—10	8.62 ± 1.26	9.25 ± 1.36	-0.63 (-5.67 – 4.42)	0.792	0.006	0.062
gait velocity (m/s)	6-months	11—10	1.42 ± 0.35	1.27 ± 0.36	0.16 (-0.27 – 0.58)	0.439	0.051	0.165
step length (cm)	6-months	11—10	70.04 ± 8.80	65.35 ± 9.04	4.69 (-5.97 – 15.35)	0.357	**0.071**	0.215
gait velocity_CV_ (%)	6-months	11—10	4.10 ± 1.40	2.30 ± 1.44	1.80 (0.11 – 3.49)	0.039*	**0.309**	0.803
step length_CV_ (%)	6-months	11—10	3.93 ± 2.21	4.33 ± 2.27	-0.40 (-3.07 – 2.28)	0.752	0.009	0.069

*Values for each group are shown as adjusted means* ± standard deviations; n: sample size; IG: intervention group; CG: control group; Diff. (95% CI): mean difference between groups (95% confidence interval); EQ-5D: EuroQol-5Dimensions; WOMAC: Western Ontario and McMaster Universities Osteoarthritis Index; STS: 30 s sit to stand test; TUG: timed up and go test; CV: coefficient of variation; rep: repetitions; * p ≤ *0.05; medium (η*_*p*_^*2*^ ≥ *0.06) and large effect sizes (η*_*p*_^*2*^ ≥ *0.14) indicated by bold text*

**Table 5:** Post-hoc sample size calculations for the different outcomes at six-months follow-up

Outcome Measures	Sample Size (A1)	Sample Size (A2)
EQ-5D Index	43	75
EQ-5D health status	53	31
WOMAC pain	75	52
WOMAC stiffness	111	485
WOMAC function	100	165
STS	707	n/a
TUG	76	1302
gait velocity	274	148
step length	265	105
gait velocity_CV_	18	21
step length_CV_	597	866

*Sample size calculations (test family* = *F-tests, statistical test* = *ANCOVA, type of power analysis* = *post-hoc, α* = *0.05, sample size* = *21, numerator df* = *1, number of groups* = *2, number of covariates* = *6 or 7) based on the found effect sizes for the different outcomes at six-months follow-up; power (1 - β error probability) threshold* = *0.8; A1: not accounting for number of therapy sessions; A2: number of therapy sessions accounted for; EQ-5D: EuroQol-5Dimensions; WOMAC: Western Ontario and McMaster Universities Osteoarthritis Index; STS: 30 s sit to stand test; TUG: timed up and go test; CV: coefficient of variation*

## Discussion

The aim of this prospective randomized controlled pilot study was to assess the effect of a digital-assisted group therapy concept on subjective outcomes, strength of the lower extremities, and functional mobility as well as gait parameters in patients with total hip and knee arthroplasty at three- and six-months follow-up. The main findings were: (i) the IG received significantly more therapy sessions than the CG; (ii) at three-months follow-up, the WOMAC stiffness score and the CV of gait velocity were higher in the IG compared to CG; (iii) at six-months follow-up, quality of life (i.e., EQ-5D index, EQ-5D health status) was higher in the IG along with a lower pain, stiffness, and better physical function (WOMAC dimensions) as well as TUG performance. Moreover, the CV of gait velocity was significantly higher in the IG compared to CG. When accounting for the differences in the number of therapy session in the statistical analyses for the six-months follow-up, (iv) the IG had still a higher EQ-5D index and EQ-5D health status as well as a lower WOMAC pain score. Moreover, step length and the CV of gait velocity were higher in the IG compared to the CG, when the results were adjusted for the number of therapy sessions.

According to the verbal inquiry, the IG received significantly more therapy sessions during the first six postoperative months compared to the CG. In general physiotherapy proved to be effective for improving clinical outcomes after total hip and knee arthroplasty [7, 8, 33]. However, postoperative rehabilitation programs differ in type, length, and cost effectiveness. It has been reported that group and individual physiotherapy show similar clinical outcomes with an economic advantage for group therapy [15, 34]. The novel digital-assisted therapy concept, which was evaluated in the present study, combines the advantages of a group therapy with an individual therapy progression based on the patient ‘s condition. The approach allows the supervision of up to five patients by one therapist leading to less time expenditure per patient.

The results of the present study revealed only small differences in several outcomes between groups at three-months follow-up. These data collectively indicate that the digital-assisted rehabilitation approach is in short term after total knee and hip arthroplasty similarly effective as the standard physiotherapy that depends on the prescription of the supervising physician. This is in agreement with Wijnen et al. showing comparable functional outcomes after twelve weeks of app-based therapy at home and standardized outpatient rehabilitation [14].

Despite the minor differences in all outcomes between groups at three-months follow-up, quality of life (i.e., EQ-5D index, EQ-5D health status) was higher in the IG at six-months follow-up along with a lower pain, stiffness, and better physical function (WOMAC dimensions) as well as TUG performance. Given that the IG received more therapy sessions, these results can be partly explained by the higher training volume. However, when accounting for the number of therapy sessions in the statistical analyses, the IG had still a higher quality of life (i.e., EQ-5D index, EQ-5D health status), a lower WOMAC pain score, and an increased step length. These data indicate that the novel digital-assisted therapy concept might induce better mid- and long-term outcomes compared to the standard physiotherapy in Germany. Although not directly transferable, a recently published systematic review on the comparison of telerehabilitation and face-to-face rehabilitation after total knee arthroplasty points in the same direction. The authors have found that telerehabilitation induced a similar pain relief, a better WOMAC score as well as higher range of motion in extension and quadriceps muscle strength [35]. The higher quality of life and the lower WOMAC pain score of the IG might be due to the regular therapy routine consisting of two sessions per week as well as the social support generated by the group setting. This is supported by the data of Lenguerrand et al., who have shown that group therapy improved patient satisfaction twelve months after knee arthroplasty along with functional outcomes [36]. In general, social support appears to be positively associated with patient-reported outcomes after joint replacement [37, 38]. Given that health-related quality of life is an important and all-encompassing outcome, which is often used to investigate surgical and rehabilitation outcomes after total knee or hip arthroplasty [39–41], our results are of high relevance for clinical practice.

Overall both groups improved the STS and TUG performance during the rehabilitation process as shown by previous studies [42–44]. No group differences were found regarding strength of the lower extremities measured with the STS test but higher TUG performance of the IG six months after joint replacement suggests better functional mobility. Similarly, Bade et al. reveled an improvement in TUG performance after eleven weeks of high-intensity (i.e., progressive resistance exercise) as well as low-intensity rehabilitation (i.e., slow transition to weight-bearing exercises and less progression in difficulty) following total knee arthroplasty [43]. Even though the training modality differed (i.e., high- vs. low-intensity), the number of therapy sessions was equal between groups leading to similar increases in TUG performance. In comparison to that, the higher improvement of the IG in the current pilot study could be related to the higher number of therapy sessions in the first postoperative six months, since the medium effect was only observed when not accounting for the number of therapy sessions in the statistical analysis.

The conducted gait analyses revealed an increase in step length for both groups but a higher step length in the IG six months after total hip and knee arthroplasty, when adding the number of therapy sessions to the statistical model. Previously published studies revealed increased stride or step length during the rehabilitation process after total knee or hip replacement, but patients did not reach stride or step length of a healthy population even one year after surgery [45, 46]. Therefore, an increase in step or stride length during the rehabilitation process seems to be a desired outcome. Although the increase in gait velocity was similar for both groups, which is consistent with the findings in the literature [47], the CV of gait velocity was significantly higher in the IG compared to the CG at six-months follow-up, even when controlling for the number of therapy sessions. Even though the number of hip and knee joints was considered in the statistical analysis, the different and extensive side effects after total knee and hip joint arthroplasty could have influenced the postoperative outcomes such as gait performance. Literature has shown that the short- and long-term clinical outcomes vary between patients undergoing knee or hip replacement [48]. An additional explanation for these results can be found in the intervention design. While the novel digital-assisted individualized group therapy concept focused mainly on strength, balance, and joint function, the individual physiotherapy for patients of the CG might have included specific gait training. A review by Hausdorff indicated that an impaired gait velocity, stride length, and especially gait variability are associated with fall risk in the elderly population [49]. Therefore, the higher CV of gait velocity observed in the IG compared to the CG might indicate a higher risk of falling. However, the CV values were small for both groups in comparison to those reported for older males (5.0% ± 2.9%) and females (5.6% ± 3.4%) aged between 70 and 74 years [50]. Consequently, even though the risk of falling seems to be small, the novel digital-assisted therapy concept could be improved by adding exercises focusing on the reduction of gait variability. Moreover, the CV of gait velocity is dependent on environmental factors and the methodology of the walking test [51]. Maximal gait velocity has been shown to be a better predictor for the short-term functional recovery after total hip arthroplasty than the self-selected walking velocity [52] and should therefore be used in future clinical trials to reduce the CV of gait parameters.

There are some limitations in our pilot study. The sample size of 26 is relatively small and therefore only small group differences were expected. However, positive trends in the IG were observed indicating an equal and partially higher effectiveness in comparison to the standard physiotherapy in Germany following hip or knee replacement. Moreover, two dropouts were reported in the IG, but those occurred prior to the start of the intervention. During the intervention process no study participant dropped out, suggesting a high adherence of patients despite the high time expenditure of therapy twice a week for several months. Although no significant group differences were found regarding anthropometric measurements, sex, or affected joint (hip or knee), even small differences could have affected the outcome of this study. We addressed this limitation by adding those variables as covariates to the ANCOVA. Based on the post-hoc sample size calculations, a full-sized randomized controlled trial would require at least 75 participants to be enrolled to reach a power of 80% for quality of life (EQ-5D) six months postoperatively. Furthermore, future studies should more accurately verify the time and cost efficiency of the novel digital-assisted and individualized group therapy concept.

## Conclusion

The novel rehabilitation concept offers the possibility to address the individual needs of the patient by implementing digital devices in a group therapy and to optimize the physiotherapists’ workforce. The results of this pilot study indicate that the novel therapy concept had a positive effect on clinical and functional outcomes after a six-months intervention period and that it is worth to conduct a larger trial to determine the efficacy and the economic advantages of the novel therapy concept further.

## Supplementary Information

**Additional file 1.** **Appendix** - Additional file 1 contains a list of physiotherapy exercises performed during the intervention. It is divided into therapy session procedure, warm-up exercises, main exercises following hip or knee arthroplasty, and cool-down exercises. 

## Data Availability

The datasets used and analyzed during the current study are available from the corresponding author on reasonable request.

## Abbreviations

ANCOVA: Analysis of covariance
BMI: Body mass index
CG: Control group
CI: Confidence interval
CONSORT: Consolidated Standards of Reporting Trials Statement
CV: Coefficient of variation
EQ-5D: EuroQol-5Dimensions
ICD-10: International Statistical Classification of Diseases and Related Health Problems
IG: Intervention group
OA: Osteoarthritis
STS: 30 s sit to stand
TUG: Timed up and go
WOMAC: Western Ontario and McMaster Universities Osteoarthritis Index
