# Supplementary material for: The effect of a digital-assisted group rehabilitation on clinical and functional outcomes after total hip and knee arthroplasty—a prospective randomized controlled pilot study
Source: BMC Musculoskelet Disord. 2023 Mar 14;24:190. doi: 10.1186/s12891-023-06270-8 (PMC10010966; doi:10.1186/s12891-023-06270-8)
Supplement: Supplementary file 1 — Additional file 1. Appendix - Additional file 1 contains a list of physiotherapy exercises performed during the intervention. It is divided into therapy session procedure, warm-up exercises, main exercises following hip or knee arthroplasty, and cool-down exercises. [file 12891_2023_6270_MOESM1_ESM.pdf]

Appendix - Additional file 1

Table S1: Therapy session procedure

	Number of exercises
**Warm-up**	2 exercises (Tab. 2)
**Main exercises**	4 exercises, one from each therapy block (Tab. 3 or Tab. 4)<br>organized as circuit training with 4 YOLii monitors
**Cool-down**	2 exercises (Tab. 5)

Table S2: Warm-up exercises

Weight shift right / left
Weight shift front / back
Steps on the spot
Toe stand
Heel stand
Walking on the spot while lifting the heels
Walking on the spot while lifting the knees

Table S3: Main exercises for patients following hip arthroplasty

	resistance band	core stability / floor	stepper	balance pad
**basic**	hip extension, standing position	modified dead bug, arms isolated, heels on floor	tap on stepper	weight shift on balance pad
**advanced**	higher resistance	modified dead bug, legs 90° flexed in the air	stand on tiptoes	
**basic**	hip flexion, standing position	flex and stretch legs in supine position	step ups	steps on balance pad
**advanced**	higher resistance	supine toe taps	step before and behind	
**basic**	hip abduction, standing position	bridging	step side to side	repetitive one-legged stand
**advanced**	higher resistance	modified bridging, one leg stretched	jump side to side	maintain one-legged stand
**basic**	hip adduction, standing position	hip abduction in supine position	dynamic hip flexion and extension	squat on balance pad
**advanced**	higher resistance	hip abduction in lateral position		

Table S4: Main exercises for patients following knee arthroplasty

	weight cuff	core stability / floor	stepper	balance pad
**basic**	hip abduction and adduction, standing position	modified dead bug, arms isolated, heels on floor	tap on stepper	weight shift on balance pad
**advanced**	more weight	modified dead bug, legs 90° flexed in the air	stand on tiptoes	
**basic**	knee flexion and extension, standing position	one-legged cycling in supine position	step ups	steps on balance pad
**advanced**	more weight	cycling in supine position	step before and behind	
**basic**	hip flexion and extension, standing position	bridging	step side to side	repetitive one-legged stand
**advanced**	more weight	modified bridging, one leg stretched	jump side to side	maintain one-legged stand
**basic**	repetitive one-legged stand	crunches	dynamic hip and knee flexion and extension	squat on balance pad
**advanced**	more weight	Side crunches		

Table S5: Cool-down exercises

Stretching of the adductor muscles
Stretching of the hamstring muscles
Stretching of the quadriceps muscle
Stretching of the calf muscles
